# Barriers and opportunities for enhancing patient recruitment and retention in clinical research: findings from an interview study in an NHS academic health science centre

**DOI:** 10.1186/1478-4505-13-8

**Published:** 2015-03-12

**Authors:** Mary Adams, Louise Caffrey, Christopher McKevitt

**Affiliations:** King’s College London, Division of Health and Social Care Research, and National Institute for Health Research (NIHR) Biomedical Research Centre at Guy’s & St Thomas’ NHS Foundation Trust and King’s College London, Faculty of Life Sciences and Medicine, Capital House, 42 Weston Street, London, SE1 3QD UK

**Keywords:** Academic Health Science Centres, Barriers and opportunities to patient recruitment, Clinical research subjects, Clinical research teams, Interface of clinical research and care, Clinical research awareness, NIHR recruitment to research policy, Patient recruitment to clinical research, Personal research benefit, Qualitative research

## Abstract

**Background:**

In the UK, the recruitment of patients into clinical research is a national health research and development policy priority. There has been limited investigation of how national level factors operate as barriers or facilitators to recruitment work, particularly from the perspective of staff undertaking patient recruitment work. The aim of this study is to identify and examine staff views of the key organisational barriers and facilitators to patient recruitment work in one clinical research group located in an NHS Academic Health Science Centre.

**Methods:**

A qualitative study utilizing in-depth, one-to-one semi-structured interviews with 11 purposively selected staff with particular responsibilities to recruit and retain patients as clinical research subjects. Thematic analysis classified interview data by recurring themes, concepts, and emergent categories for the purposes of establishing explanatory accounts.

**Results:**

The findings highlight four key factors that staff perceived to be most significant for the successful recruitment and retention of patients in research and identify how staff located these factors within patients, studies, the research centre, the trust, and beyond the trust. Firstly, competition for research participants at an organisational and national level was perceived to undermine recruitment success. Secondly, the tension between clinical and clinical research workloads was seen to interrupt patient recruitment into studies, despite national funding arrangements to manage excess treatment costs. Thirdly, staff perceived an imbalance between personal patient burden and benefit. Ethical committee regulation, designed to protect patients, was perceived by some staff to detract from clarification and systematisation of incentivisation strategies. Finally, the structure and relationships within clinical research teams, in particular the low tacit status of recruitment skills, was seen as influential.

**Conclusions:**

The results of this case-study, conducted in an exemplary NHS academic research centre, highlight current systematic challenges to patient recruitment and retention in clinical studies more generally as seen from the perspective of staff at the 'sharp end’ of recruiting. Staff experience is that, beyond individual clinical research design and protocol factors, wider organisational and extra-organisational norms, structures, and processes operate as significant facilitators or hindrances in the recruitment of patients as research subjects.

## Background

Given recent UK national policy directives encouraging a 'research embedded National Health Service (NHS)’ [1], biomedical research is an important organisational priority for all NHS organisations. In addition, a key recommendation of the government’s Life Science Strategy is for health care organisations to respond to “*the growing readiness of patients to participate in research*” and to provide adequate infra-structural support to researchers to respond to patient interest and choice with respect to research participation [2]. Nevertheless, there is extensive research evidence from the NHS acute hospital sector that poor patient recruitment or retention of patients to clinical research is widespread, leading to delays in the start or completion of both academic and commercially funded research, the reduced external validity of studies, and wasted public resources and opportunity for patient participation [3–6].

Studies examining the factors that support or detract from the successful recruitment and retention of patients into clinical studies tend to focus on the influence of one or more of the following three interconnected factors.

First, procedural issues; research suggests that methods of patient recruitment into individual or into a series of research studies can influence recruitment [3, 7]. This includes the finding that business models to promote research participation can aid recruitment [7, 8]. In addition, more successful recruitment rates and higher positive research awareness have been associated with multi-disciplinary teams’ involvement in recruitment [6] and with wider stakeholder involvement in research design and processes [6, 9].

Second, communication and perceptual issues; how the immediate and longer-term value of clinical studies [5, 10, 11], as well as specific outcomes of a study [12], are perceived by and communicated to research participants, other patients, the public, and the clinical and clinical-research communities. Patients’ perceptions of the harm or benefit of studies have been found to be influential [13, 14], as have their treatment preferences [14–16]. However, the literature suggests that these perceptions do not exist in isolation. Rather, the quality of a clinical relationship or encounter is noted as significant for engaging patients as research subjects [17, 18] and for retaining them as such [19]. It has been found that patients’ acceptance of equipoise can be unintentionally influenced by how the research is communicated to them [18, 20, 21]. In addition, participation can be influenced by the ability of recruiters to explain complex trials [22–24]. More broadly, studies have asserted the need for improved public and patient awareness of clinical research [5, 6, 25] and clinical research outcomes in general [12]. The 'public and patient participation’ approach is recently echoed in National Institute of Health Research (NIHR) policy initiatives [26] – where it is anticipated that the more patients ask about research, the more NHS staff will become involved in research – and in directives from the Association of Medical Research Charities [27], which notes the recruitment potential of patients’ self-referring into studies and bypassing their own clinicians.

This self-referral approach is based also on the finding that patients themselves are not the only gatekeepers to recruitment. The influence of clinical and non-clinical 'gate keepers’ within clinical services has also been identified [5, 25, 28–30] and the literature emphasises the impact of communication of the research to recruiters. In a systematic review of the literature, Ross et al. [15] found that clinicians’ perceptions of the importance of the research question impacts recruitment. Similarly, it has been found that recruiter perceptions of potential harm or benefit to patients are important [5]. Research has further explored the potential for recruiters to be less willing to approach potential participants due to recruiter misunderstandings regarding aspects of the trial [14, 22, 31, 32].

Third, resourcing issues; how national and organisational incentivisation strategies stymie recruitment work within, and between, individual studies. Proposed strategies to enhance this work include reducing clinicians’ clinical workload [5] and incentivisation of clinical staff without clinical research remits, for example, the provision of protected time, training, or career progression opportunities [5, 7, 25, 33]. In addition, several studies highlight the advantages of investment in cross-project, cross-service, or cross-organisational recruitment databases or 'gate ways’ for sustaining pools of potential research subjects [34, 35].

Despite this extensive literature on strategies for enhancing patient recruitment to research, gaps remain. The current literature base rightly emphasizes the success of national, systematic initiatives, including the NIHR, Clinical Research Network, to improve recruitment [36, 37]. However, to the authors’ knowledge, the literature has not, to date, assessed whether factors at the national or systemic level can simultaneously and unintentionally present barriers to recruitment in particular research contexts. In addition, the influence of organisational context on recruitment, as well as the importance of understanding recruiters’ perceptions, has increasingly been recognised. More recently, the value of qualitative studies has been highlighted with a view to addressing these concerns [5, 7, 8, 21, 22, 38]. In particular, Fletcher et al. [5] briefly draw from established organisational sociology concepts and models to examine the variously levelled reasons for poor patient recruitment at patient, recruiting clinician, trial design, trial centre, and organisational levels. Therefore, despite the extensive literature on strategies for enhancing patient recruitment to research, there remains a lack of substantive, high quality research evidence of the actual activities and experiences of patient recruitment work [5, 8, 38] and of the impact of organisational strategies on this work. Moreover, as Gul and Abi [39] note, the majority of recommended interventions for enhancing the recruitment and retention of patients in clinical studies are 'piecemeal’ (for example, the improvement of patient information resources) and take little account of how local cultures and practices of recruitment work influence the effectiveness of such interventions. In other words, it is unclear how, and how far, the different sets of issues that support or undermine patient recruitment into research are interconnected in general and specific clinical settings, research teams, and research centres.

In order to contribute to filling these gaps in the literature, this study sought to explore perceived barriers and facilitators to patient recruitment and retention to clinical studies from the perspective of staff that carried immediate responsibility for this work. This article also adopts a specific focus on trust and extra-organisational or national level factors that influence recruitment in this context [37]. While the study took place in one clinical research group in an Academic Health Science Centre (AHSC), the theoretically generalised findings suggest the significance of trust and national level factors. Given that the factors we identify are by no means unique to this setting, their perceived significance in this context suggests they are likely to be relevant beyond the immediate context, with wider implications for clinical research policy in the UK and internationally.

## Methods

The study was conducted in one clinical research group^a^, comprising several research teams, in a large inner-city teaching hospital (acute trust) operating as a secondary and tertiary referral centre. The hospital forms part of one of six AHSCs in England and thus subscribes to a vision of improved patient care, achieved through an NHS and university partnership providing co-located research, health education, and hospital services. The acute health trust, was nationally acclaimed as an organisation “*leading the way in opportunities for patients to take part in clinical research studies*” [unpublished personal communication] and ranked in the national top 10 trusts for both the quality of its clinical research and the number of hospital patients involved in a clinical study [40]. Nevertheless, based on regular monitoring of research recruitment processes, local research and development managers considered that some clinical research groups were underperforming in relation to patient recruitment. The clinical research group in this study (one of 12 groups organised around different clinical research groupings in the Trust) fell into that category. During the year of the study (October 2011 to September 2012) its recruitment figures represented 3.5% of all patients recruited to research in the Trust, and, in some instances, trials had been open for one or two or more years without recruiting one suitable research participant.

Following ethical overview and Research and Development approval of the study by the acute hospital trust, all research teams in the clinical research group were notified of the objectives of the study. All research staff invited to interview were again notified of the objectives of the study and of the purpose of the interview. Each potential interviewee was assured, in writing, of their entitlement to anonymity and confidentiality. Written consent was acquired from each interviewee and each interviewee was reminded of their entitlement to withdraw their data from the study database for up to three months after their approval and return of their own interview transcript.

The data were collected between December 2011 and March 2012. Initially, purposive sampling of a range of key staff in research teams (including chief and principal researchers, research fellows, and support staff) involved in patient recruitment (n = 15) was initiated under the guidance of local Research and Development leads. Due to the limited or non-response of these staff, subsequent snowball sampling was conducted on the advice of these key staff. Sampling was intended to identify those staff with greater experience or expertise in patient recruitment work rather than to capture a numerically representative sample of informants [41]. Overall, 15 staff were invited to interview and 12 accepted the invitation. The work roles of interviewees were: Trial Facilities Manager (n = 1), Senior Research Nurse (n = 1), Clinical Research Fellow (n = 2), and Research Nurse (n = 7). Interviewees who were clinical researchers (n = 2) were junior staff. The research nurses were also senior clinicians (clinical nurse specialists and/or research team leads), however, their professional assistance or exchange networks were defined by the Principal Investigators and Chief Investigators on the studies that they were responsible for recruiting to. All senior medical researchers within the clinical research group were approached to take part in this research. However, they deferred this responsibility to nurses in their teams because they considered these staff responsible for patient recruitment.

Open-ended, one-to-one interviews were conducted with all recruited staff and were guided by two broad research questions. After establishing the work role, organisational position, and work experience of each interviewee, interviewees were asked their views on what factors either support or undermine the recruitment of patients into clinical research in this research group and in the studies that they were responsible for in particular. Eleven staff interviews were conducted at work in private office rooms and lasted between 40 and 70 minutes. One of these interviews was conducted by telephone in a private office space and lasted 35 minutes. Interviews were conducted by one social scientist (MA), who took short notes at the interview, typed up longer notes immediately after the interview, and returned these notes to interviewees within 24 hours for corrections and additions before entering the anonymised transcripts into a password-protected research database. Notes were taken because the research was originally conducted as an initial mapping exercise of issues in the clinical research group. The interview schedule was limited to two core questions: i) what factors support the recruitment of patients into clinical research here and ii) what factors inhibit the recruitment of patients into clinical research here. The interview schedule was deliberately open to allow for interviewees own interpretation of factors. Probes were as neutral as possible, e.g., 'can you tell me a little bit more about this?’ 'Can you give me an example?’ One transcript was not returned and so was removed from the database; therefore, data were collected from 11 interviews. Returned transcripts were organized and coded by MA using qualitative data analysis software (NVivo9).

Following Ritchie, analysis of the data involved, first, a descriptive examination “*unpacking the content and nature of a particular phenomenon or theme*” [42] from the data set and, second, an explanatory account that involved “*finding links and connections between two or more phenomena*” and that offered “*a deeper understanding of the subject under review*” [43] Thus, our descriptive account summarised key limiting and facilitating factors to patient recruitment, as noted by the 11 interviewees, and our explanatory account sought to understand these factors more systematically. MA undertook the initial literature review, data analysis, and write up, and CM checked this by reading a random sample of the transcripts. LC added further literature and supported MA with the final write-up. In addition to adhering to established procedures to anonymise interview transcripts, care was taken to ensure that opinion given at interview could not be attributed to a single individual to protect confidentiality.

## Results and discussion

The researcher (MA) made approaches to all senior researchers (Chief and Principal Investigators) responsible for clinical studies that were 'open’ (recruiting patients) during that period. These studies ranged from emergency and elective surgical procedures, drug studies, remote monitoring and imagining, and exercise studies. While our interviewees noted that different studies presented different challenges for recruitment, they all independently volunteered the view that patient demographics (particularly the challenges of recruiting elderly patients into research), anticipated costs and benefits (travel vs. additional screening for example), and organisational factors, were more significant to recruitment success than the study itself.

Our descriptive account identified staff views on the key factors that facilitated or undermined the recruitment and retention of patients into research. The data were also sorted and synthesized by a thematic chart to summarise what staff discussed as the location of these key factors (at patient, research-study, team, centre, organisation (trust), and extra-organisational levels). This descriptive account of key factors, their organisational location and their direction of influence, along with the frequency of staff views on this, is summarised below.

### Summary of staff views on key barriers and opportunities for enhancing patient recruitment to clinical research and their location

A. **Competition for research participants**

*Study-Specific Factors*

- Demands of study protocols (multiple exclusion criteria) (11/11 interviewees)
- Clinical populations with complex chronic disease (9/11)
- Competition with similar studies (8/11)

*Centre-Specific Factors*

- Presence of research teams requiring similar clinical populations (7/11)

*Trust-Specific Factors*

- Competition between departments/divisions for patients for own 'portfolio’ studies (6/11)

*Extra-Organisational Factors*

- League tables and 'pay for performance’ raises competition for research subjects (5/11)
- Insufficient incentivisation of collaborative referrals of patients into research (4/11)

B. **Interface between clinical research and clinical care**

*Team-Specific Factors*

- Proximity of team to clinical areas (build research 'presence’ and relationships) (7/11)

*Trust-Specific Factors*

- Non-research staff have limited resources to undertake research tasks
- Value of clinical research not established/recognised in clinical areas

*Extra-Organisational Factors*

- Limited resourcing to raise general awareness of research to patients and the public (7/11)

C. **Patient costs and benefits**

*Patient Specific Factors*

- Need to protect older/more vulnerable patients from excessive research burden (8/11)

*Team-Specific Factors*

- Availability of staff providing 'personal benefit’ from participation (7/11)
- Relations of mutual reciprocity between staff and participants (6/11)

*Trust-Specific Factors*

- Access to additional extra service resources for participants (9/11)

D. **The Clinical Research Team**

- Commitment of research team to the particular study (6/11)
- Availability of staff providing 'personal benefit’ from participation (7/11), as above
- Relations of mutual reciprocity between staff and participants (6/11), as above
- Proximity of team to clinical areas (build research 'presence’ and relationships) (7/11), as above
- The status of recruitment work (if valued by senior research clinicians) (10/11)
- Team approach to research design (including recruitment feasibility) (6/11)
- Team approach to recruitment work (as simple task or complex skill) (8/11)

Our explanatory account highlights and examines the four key factors that influence recruitment success; these are: i) competition for research participants; ii) intersections between clinical care and research; iii) patient costs and benefits, and iv) the clinical research team. We found that the nature of the clinical research team was perceived by interviewees as a factor that could moderate several other key factors.

### Competition for research participants

The findings suggest that the focus on competition for research participants at organisational (both centre and trust) and national level can undermine the collaboration necessary for ongoing patient recruitment to different studies. Interviewees related that studies within the research group often had similar, multiple, and complex exclusion criteria. As the research centre was located in a specialist (tertiary) acute hospital trust, the patient population also had a high frequency of complex clinical conditions and treatment histories which reduced the likelihood of research eligibility. Studies were therefore recruiting from a highly limited population while eligible participants had to be identified from amongst the wider population. The difficulty of recruitment to studies with multiple and complex exclusion or inclusion criteria was clear from the anonymised 'recruitment activity sheets’ kept for larger scale clinical studies. Examination of these 2009 to 2011 records for four major studies showed that an average of one in ten patients was recruited after their consent to participate in initial screening for research eligibility.

Despite this challenging context for identifying eligible participants, there were limited systems and processes for the referral of potential research subjects between different research teams within the centre and between clinical departments. Interviewees related that research teams (their own and others) were often protective of their own 'consent to be contacted’ patient databases. For example, two extensive databases of patients willing to be involved in future studies were kept within individual research teams and not shared more widely within the centre or across the trust. Keeping ongoing and exclusive relationships with 'regulars’ was seen as an important lever for recruiting patients to forthcoming studies more quickly (so that studies met 'by time and target’ recruitment performance measures). Interviewees perceived that sharing a database risked losing these future research participants. For teams that had invested their own time (including that spent applying for the temporary funding of support staff) in building and maintaining a database that allowed them repeated access to their own 'regulars’ (patients who took part in research on a serial basis and who were already on good terms with them) the risks of collaboration in this scenario were not always seen as worthwhile.

Some interviewees noted that the lack of collaboration between research teams stemmed in part from the national policy drive to double the numbers of patients in research within 5 years. The attempt to achieve this, by publishing league tables and providing quarterly benchmarks of recruitment by trust, hospital, and centre, was considered to undermine collaborative recruitment work between organisations, research centres, and, in some cases, research teams. Despite this, the competition for patients in research did not affect all staff or research teams equally. Some interviewees referred to informal and ongoing 'knock for knock’ (reciprocal) arrangements that sometimes existed across teams, centres, and trusts. These always involved more senior and longer established clinical researchers. Senior researchers were further advantaged by their increased ability to run better-funded studies that could successfully attract more patients by investments in high quality promotional literature and regular study updates. By contrast, recruitment was perceived as especially challenging for junior researchers who lacked established reputations or established and informal working relationships. In this sense, the findings suggest that the national focus on competition to drive research may undermine collaboration in some contexts, particularly for less established researchers.

### The interface of clinical research and clinical care

The findings also highlight a perceived gap in national provision for dealing with the additional burden that research could place on clinical teams. When recruiting patients from acute and emergency care, research teams often had to rely on non-research staff to identify potential research participants for particular studies. Non-research staff did not have protected or allocated time for recruiting patients and interviewees perceived that busy ward and outpatient staff easily forget about research work. Experienced research nurses sought to address this locally by relocating their offices or clinical rooms closer to non-research clinical areas. Their closer proximity to ward or outpatient staff and patients provided time for them to build good relations for future potential referrals of patients and helped to remind and encourage busy clinicians about research activity.

Similarly, our findings from staff interviews indicate the inadequacy of national provision for dealing with additional treatment costs, which could be associated with research, or poor trust management of these costs. Staff perceived that, without such payments being met (and felt) on the appropriate front-lines of wards and clinics, there was little to incentivise non-research staff to work towards retaining patients in ongoing studies, particularly when patients moved though different organisations, services, and clinical areas over time. Notably, research participants might become ineligible for continued study participation because of the demands of routine research tasks on ward and clinic time. Thus, ward staff were known to be sometimes unable or unwilling to accommodate the additional needs of research participants (such as additional monitoring or screening procedures, on-going sample collections, or overnight admissions for pre-surgical research assessment). While national agreements exist for organisational management of 'excess treatment costs’ [27], these arrangements did not extend to ward levels. In this sense, while national funding arrangements acknowledge the excess treatment costs attributable to research, interviewees perceived that these arrangements did not fully address them.

On this basis, most interviewees questioned recent efforts, initiated at a national policy level [44], to promote 'patient-led’ enquiries for improved research participation opportunities. Indeed, several nurses commented on the perceived shortcomings of a recent national drive for a patient-led research recruitment campaign. One interviewee remarked: “*It’s fine to say 'It’s OK to Ask’… but who do they think is going to be around to give the answers?*” (Interviewee 5). The majority of interviewees viewed the challenge of improving recruitment as not about raising general patient awareness and enthusiasm about research but about the exercise of specialist clinical knowledge and clinical relationships for identifying those patients eligible for particular studies. Interviewees justified their focus on research recruitment, rather than research awareness work, in two ways, namely the need to protect patients from being rejected from studies for which they were unsuitable candidates and the need to protect their time from these unfruitful enquiries.

### Patient costs and benefits

The findings suggest that some interviewees perceived that systemic issues prevented them from addressing the burden of research for patients, with implications for patient recruitment. Interviewees agreed that many patients enter research with the desire to 'give something back’. However, some believed that in order to encourage patients to take part and to recognise their contribution to research, patients should receive personal research benefit. These staff sometimes sought to incentivise patients by stressing indirect benefits of participation, available irrespective of randomisation. These included early screening, additional clinical time, or quicker access to their consultant by informal referral by a research nurse. However, these staff felt limited in what they could formally offer to research participants because of the regulatory demands of ethics committees on research with patients. Patient information has to promise to offer no assurance of personal benefit to patients in research in order to protect the principle of informed, freely-given consent (without financial inducement). Staff felt that this emphasis on the lack of personal benefit (particularly when patients as well as funding bodies got personal benefit as clinical benefit) acted as a barrier to recruiting patients who expected sufficient compensation for, and recognition of, their time and clinical labour. In this sense, established approaches of national ethics committees were viewed by some interviewees as a barrier to the appropriate incentivisation or compensation of patients in research.

In keeping with previous research [5, 7, 15], the findings also emphasise the importance of ensuring that research is not perceived by clinicians to place an excessive burden on patients or to compromise their safety, and that research has clear benefits for patients. More experienced staff noted their professional responsibility to assess and manage the cost/benefits of research participation for patients through the course of a study. It was generally agreed (by research fellows and nurses) that research nurses were better positioned to assess the cost/benefits of research compared to senior clinical academics (who were less involved in the day-to-day running of studies) or patients themselves (who had to rely on narrow or generalised patient information literature). These research nurses were often more critical of the excessive research burden that they perceived some on-going studies placed on patients. In addition, many staff privately questioned the ethical justification for some on-going AHSC studies, particularly long-running studies with future results that had already been overtaken by changes in (as yet unproven) clinical practice. Such studies often 'lagged’ in recruiting patients because staff were unsupportive of the study, particularly when faced with opposition from non-research clinical colleagues. The findings also suggest that staff perceived that schedules of return hospital visits, home monitoring requirements, lifestyle changes, or treatment regimens often created unexplained or unanticipated burdens of research participation that were too onerous for the elderly or the very sick. However, other staff noted the dangers of anticipating that the elderly and vulnerable should be excluded from a choice to participate in studies.

In this sense, the findings suggest the criticality of ensuring that research, funded by national bodies on the basis of the important contribution that it will make and its ethical justifiability, is perceived in the same way by those on the ground. Our findings suggest that in some cases there is a disconnect between the national level and the 'sharp end’ in this regard, with implications for recruitment and retention of patients to research.

### The clinical research team

The normative organisation of clinical research teams, in terms of professional management hierarchies that separate research recruitment work from research leadership, was also found to influence patient recruitment. The research nurses and junior researchers interviewed related that, in their experience, senior clinical researchers and particularly medical or surgical consultants were more successful at recruiting patients than all other staff. Interviewees considered that this was either because patients felt less able to refuse their invitations or felt more confident of research promoted by them. In addition, as noted above, senior staff had established networks which they could use to recruit patients from across clinical areas. However, in this clinical research group recruitment to research was perceived as lowly work in contrast to other research activities. Thus, senior staff rarely recruited patients to studies themselves. Indeed, as our description of study methods above suggests, senior researchers declined our invitation to be interviewed on patient recruitment work because they did not consider this to be their work responsibility. Thus, clinicians with less authority (who, interviewees noted, sometimes have limited insight into the nature and risks of research study) were left to explain the value of participation to patients.

Our interviewees felt that this distancing of research leaders from recruitment work negatively affected recruitment. Most interviewees noted that for a study to be successful, the whole research team needed to be involved in study design, progress monitoring, and review. Interviewees suggested that a situation of shared knowledge, interest, and investment in a study enhanced successful patient recruitment in itself. In the words of one research nurse, “*successful research relies on people caring about the study… about them really believing in it*” (Interviewee 5). In this sense, an overly rigorous or exclusive division of labour within a research team may have a negative effect on recruitment. In addition, it is notable that in some other clinical research groups within the trust (and particularly in those more successful in recruiting patients in research), senior medical and nursing clinicians actively recruited patients into the studies where recruitment was most difficult and mentored junior researchers in recruitment skills. Thus, the traditional division of labour was not insurmountable but required a levelling of hierarchies to combine research leadership with research recruitment.

## Conclusions

In this study, we examined, from the perspective of staff responsible for recruiting patients into clinical research, the multi-levelled and complex nature of factors that affected work at the 'sharp end’ of practice. The research took a small scale and in-depth approach, investigating work in one clinical research group located in a research centre with an exemplary national reputation.

In some respects, the findings substantiate those of previous research, outlined in the introduction to this article. This includes the finding that, in terms of procedural issues for recruitment, inter-professional cooperation is important for the identification, recruitment, and retention of patients [3, 7, 37]. Our findings add weight to previous research, which suggests that staff views on the balance of costs against and benefits to patients in research is vital to ensure successful recruitment work [5, 7]. It is noted that these perceptions may be influenced by how the research is communicated to and understood within staff teams [14, 22, 24, 31], but this was not a focus of our study, which did not observe trial meetings. Further, our findings concur with previous research which has found that strain on clinical resources and time is detrimental to research [5, 7, 25, 33].

At the same time, rather than necessarily contradicting the findings of previous research, this article draws attention to previously overlooked issues by highlighting the need to consider how 'high level’ factors and processes get played out 'on the ground’, often in unpredicted ways. Our findings suggest that cooperation between professionals may be discouraged by the national drive to increase recruitment through competition, benchmarking, and 'payment by [recruitment] results’. While cooperation exists, this may be despite rather than because of such national initiatives. More junior staff, who have not yet built a reputation, are, in particular, not helped by the focus on competition. We suggest that further research is required to extend our understanding of the potential for auditing systems to impact recruitment in unintended ways.

In the same vein, our findings highlight that, while national funding agreements acknowledge the extra treatment and time costs attributable to research, the experience of at least some front-line staff is that these contributions are either insufficient (and so require a closer factoring into research design and funding applications) or that these contributions are not reaching the relevant clinical areas. In either case, the insufficient contribution to the research work conducted in wards and clinics leads to strain between clinical and clinical research work and resourcing priorities, with a deleterious impact on recruitment.

Maintaining the focus on previously overlooked 'high level’ factors, our findings highlight that some recruiters to research perceive that established ethical regulatory expectations – that prevent researchers from promising direct benefits to patients in research – are a barrier to being able to offer appropriate compensation and encouragement for patients offering time and clinical labour. These findings cannot provide any estimate of the prevalence of this view and, regardless, ethical standpoints are not a simple matter of democratic decision making. However, ethical standards are not static. As social constructs, they change over time. In this regard we suggest that the voices of those undertaking recruitment be heard as part of a wider critical review of the ethical requirement that patients should not receive direct benefit for participation in publically funded research.

Finally, the findings suggest that the established hierarchies of research work, with the lowly work of recruitment and retention undertaken by more junior professionals and, increasingly, non-professionals, operates as a barrier to successful work. Our interviewees noted the relative success of some senior research clinicians in recruiting patients into studies either because of their authority, knowledge of the study, or communication skills. More significant, the value of clinical research team leadership that included a whole team approach to patients in research was noted by our interviewees as an especially successful way of enhancing patient recruitment and retention.

Our study is limited in several respects. Our aim was not to examine all issues impacting recruitment. Instead, we sought to examine how staff who recruited patients understood and explained the barriers and opportunities to this work. We examined how these front-line activities are affected by policy drivers operating at national, organisational (hospital trust), and extra-organisational levels. We note that the factors identified by our interviewees are inevitably influenced by their subject positionality (for example, interviewees might emphasise systemic factors rather than focus on their own or their colleagues shortcomings). Nonetheless, the aim of this study was not to present an objective assessment of barriers affecting research, but to examine the subjective accounts of those working at the frontline of recruitment. We argue that the subjectivity of these accounts is what makes them valuable since the ways that those at the front-line of recruitment perceive their work is likely to affect their behaviour.

In terms of the research scope, it should also be noted that only the views of nominated staff representatives (those front-line and junior staff with a recruitment work) from one clinical research group in an AHSC were examined. The study was further limited to recruitment to biomedical research (and particularly clinical trials) in an acute hospital setting, rather than to health research in general. Further research in other contexts is required to examine the particular barriers and opportunities to patient recruitment to health research in other settings. Moreover, the views of non-research clinicians and patients/study participants themselves were not addressed in our study, which focused explicitly on the views of staff recruiting and retaining patients. Clinical research, including recruitment activity, is co-constructed and examination of these voices is necessary for further understanding of the issues raised in our study.

Finally, the study is limited by the small sample size, although the findings are not invalidated by this since qualitative research seeks theoretical rather than statistical generalizability [41]. However, further themes may have arisen in a larger and more diverse sample. We argue that the factors identified in this study are by no means unique to this setting and so are generalizable beyond this study. Their perceived significance in this context suggests that they are potentially perceived and experienced in similar ways in other settings, with implications for recruitment and retention to research.

## Endnote

^a^ This is common medical field, but in order to protect participants’ anonymity we have not named the specific clinical area.

## Abbreviations

AHSC: Academic Health Science Centre
NHS: National health service
NIHR: National Institute of Health Research.
